# CD40-mediated HIF-1α expression underlying microangiopathy in diabetic nerve pathology

**DOI:** 10.1242/dmm.033647

**Published:** 2018-04-26

**Authors:** Hung-Wei Kan, Jung-Hsien Hsieh, Hsiung-Fei Chien, Yea-Huey Lin, Ti-Yen Yeh, Chi-Chao Chao, Sung-Tsang Hsieh

**Affiliations:** 1 Department of Anatomy and Cell Biology, National Taiwan University, Taipei 10051, Taiwan; 2 Department of Surgery, National Taiwan University Hospital, Taipei 10002, Taiwan; 3 Department of Neurology, National Taiwan University Hospital, Taipei 10002, Taiwan; 4 Graduate Institute of Brain and Mind Sciences, College of Medicine, National Taiwan University, Taipei 10051, Taiwan

**Keywords:** Diabetic neuropathy, Microangiopathy, Ischemia, Thrombosis, Inflammation

## Abstract

To understand the pathology and molecular signatures of microangiopathy in diabetic neuropathy, we systemically and quantitatively examined the morphometry of microvascular and nerve pathologies of sural nerves. In the endoneurium of diabetic nerves, prominent microangiopathy was observed, as evidenced by reduced capillary luminal area, increased capillary basement membrane thickness and increased proportion of fibrin(+) blood vessels. Furthermore, capillary basement membrane thickness and the proportion of fibrin(+) blood vessels were correlated with small myelinated fiber density in diabetic nerves. In diabetic nerves, there was also significant macrophage and T cell infiltration, and cluster of differentiation 40 (CD40) expression was increased. The molecular alterations observed were upregulation of hypoxia-inducible factor-1α (HIF-1α), mitogen-activated protein kinase-activated protein kinase 2 (MK2; MAPKAPK2) and phosphatase and tensin homolog (PTEN). In addition, HIF-1α was correlated with small myelinated fiber density and capillary luminal area, while both MK2 and PTEN were correlated with capillary basement membrane thickness. The molecular cascades were further demonstrated and replicated in a cell model of microangiopathy on human umbilical vein endothelial cells (HUVECs) exposed to high-glucose medium by silencing of CD40, PTEN and HIF-1α in HUVECs using shRNA. These data clarified the hierarchy of the molecular cascades, i.e. upregulation of CD40 leading to HIF-1α expression in endothelium and nerve fibers. In conclusion, this study revealed the association of microangiopathy, thrombosis and inflammatory infiltrates with nerve degeneration in diabetic nerves, demonstrating that CD40 is a key molecule for the upregulation of HIF-1α and PTEN underlying the severity of microangiopathy.

## Abstract (precis)

**Summary:** This study provides a new therapeutic strategy for diabetic neuropathy, by demonstrating CD40 as a key mediator of microangiopathy leading to HIF-1α expression in endothelial cells.

## INTRODUCTION

Peripheral nerve degeneration in diabetic neuropathy, a major complication of diabetes with a frequency of 25-60%, has been attributed to multifactorial mechanisms, such as metabolic dysregulation of sorbitol, oxidative stress and neurotrophic deficiency, according to studies on experimental diabetes in rodents and human nerve pathology (Zochodne, 1999; Brownlee, 2001; Kennedy and Zochodne, 2005; Edwards et al., 2008; Bakovic et al., 2018). Although microangiopathy is listed as one of the major microvascular complications of diabetes, including reduced endoneurial capillary density and endothelial dysfunction, the cascades leading to this cellular pathology and its molecular consequences in diabetic nerves have not yet been systemically investigated.

Chronic inflammation is frequently coupled with microvascular disease in diabetes (Crook, 2004; Said, 2006). The recruitment of inflammatory cells probably occurs through hyperglycemia and glycation (Brownlee, 2001), which are also risk factors for endothelial dysfunction and thrombus formation (Grant, 2007). Inflammatory mediators can augment the expression of tissue factor genes in endothelial cells, leading to thrombosis, which, in turn, has been postulated as a cause of leukocyte adhesion and inflammatory response amplification (Libby and Simon, 2001). Among the interweaved mechanisms between inflammation and thrombosis, the cluster of differentiation 40 (CD40) signaling pathway plays an important role (Mach et al., 1997). The CD40 ligand binds to its receptor CD40 and induces tissue factor expression in macrophages and endothelial cells. However, the relationship between the CD40-CD40 ligand system and microangiopathy in diabetic nerves has remained unexplored.

Tissue ischemia, one of the major consequences of microangiopathy (Agnić et al., 2015), activates a series of signaling pathways, such as hypoxia-inducible factor-1α (HIF-1α) (Semenza, 2000). Among these cascades, both the mitogen-activated protein kinase (MAPK) family and phosphoinositol 3-kinase (PI3K; PIK3CA)/Akt pathways constitute important responses to hypoxia and regulate HIF-1α protein levels and transactivation (Emerling et al., 2005; Maynard and Ohh, 2007; Khandrika et al., 2009). The MAPK family mediates the protection of nerve fiber function and structure (Price et al., 2004; Jolivalt et al., 2011). Phosphatase and tensin homolog (PTEN), an antagonist of PI3K, is elevated during experimental diabetes and contributes to impaired regeneration of diabetic axons (Park et al., 2010; Singh et al., 2014) as well as in diabetic kidneys (Delic Jukic et al., 2018). However, the roles of the MAPK family and PI3K/Akt signaling molecules in microangiopathy have not been fully examined in human diabetic tissues. These observations raised the possibility of exploring the underlying molecular mechanisms leading to microangiopathy and its relationship with nerve pathology, either beneficial or detrimental to nerve integrity.

To address the above issues, this study aimed to investigate (1) microangiopathy and its correlation with nerve pathology, and (2) molecule signatures related to microangiopathy.

## RESULTS

### Endoneurial capillary morphometry, thrombosis and inflammatory cell infiltration

**Fig. 1.**
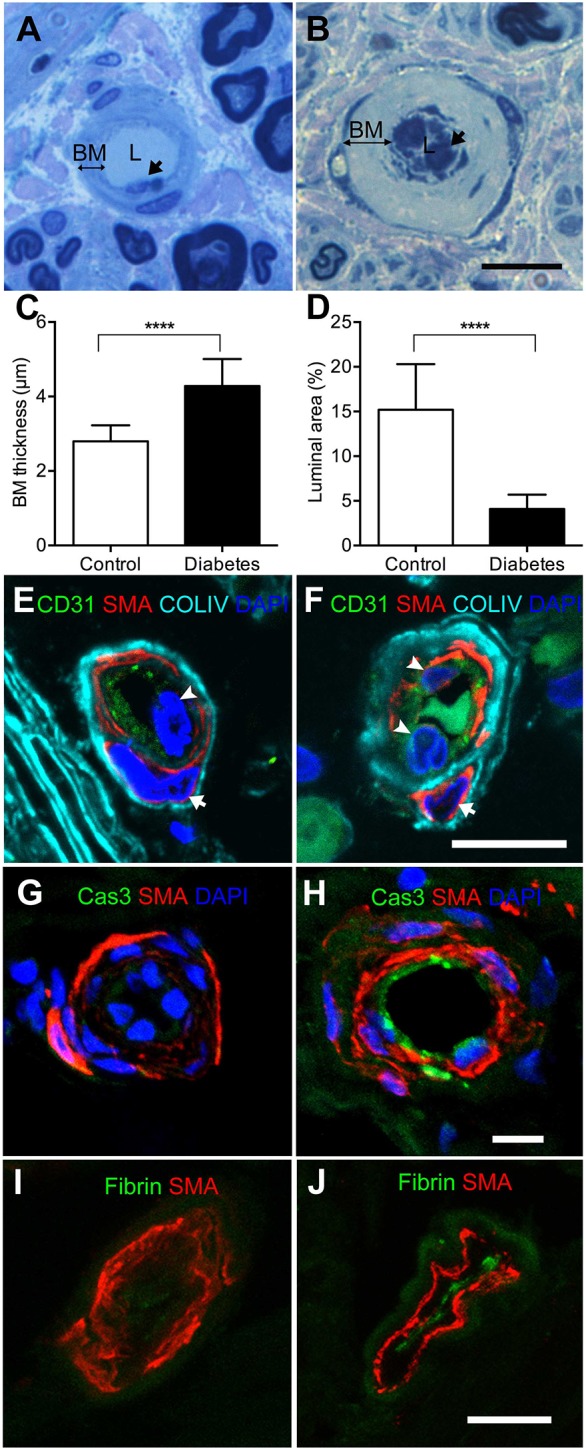
**Microangiopathy in diabetic nerves.** (A,B) Semithin sections revealed capillaries that were obviously identified by endothelial cells (arrows), lumen (L) and capillary basement membrane (BM). The endoneurial capillary of control subjects appeared normal, with appropriate capillary basement membrane thickness and adequate capillary luminal area (A). The capillary basement membrane thickness was increased in diabetes patients, with hypertrophic endothelial cells and a decreased capillary luminal area (B). (C,D) Quantification of microangiopathy by semithin sections showed significantly increased capillary basement membrane thickness and significantly reduced capillary luminal area in diabetic nerves (*n=*28) compared with control nerves (*n=*6). ****P<0.0001 by two-tailed Student's *t*-test. Data are mean±s.d. (E,F) Multiple labeling of CD31 (green), smooth muscle actin (SMA, red) and collagen IV (COLIV, cyan) was performed on endoneurial capillaries. DAPI (blue) was used for nuclear staining. Comparable to the semithin sections, the capillary basement membrane consisted of pericyte nuclei (arrows), SMA and COLIV; and the capillary basement membrane thickness was increased in diabetic capillaries (F) compared with control capillaries (E). Arrowheads indicate nuclei of endothelial cells. (G,H) Epineurial blood vessels were examined with cleaved caspase 3 (Cas3, green) and SMA (red) immunochemistry. Compared with control blood vessels (G), diabetic blood vessels exhibited increased Cas3 in endothelial cells (H), which indicated microvascular insults. (I,J) Thromboses containing fibrin clots were stained with FITC-conjugated anti-fibrin antibody (green) and endoneurial capillaries were revealed by SMA (red) immunohistochemistry. Fibrin(+) clots were absent (I), whereas there were markedly increased fibrin(+) clots (J) in diabetic capillaries. Scale bars: 10 μm.

To explore the pattern of microangiopathy, we analyzed the vascular morphometry of diabetic nerves. The pattern of microangiopathy in diabetic nerves was distinct from the vascular appearance of control nerves in which the capillary lumen was evident, with an appropriate thickness relative to the vascular basement membrane (Fig. 1A,B). In diabetic nerves, the capillary basement membrane was thicker than that of control nerves by 52.9% (4.28±0.73 versus 2.80±0.43 μm, *P*<0.0001; Fig. 1C), and the capillary luminal area was reduced by 73.1% (4.09±1.61 versus 15.2±5.12%, *P*<0.0001; Fig. 1D). However, endoneurial capillary densities were similar in both control and diabetic groups (72.4±28.0 versus 58.2±10.6 capillaries/mm^2^, *P*=0.398).

To identify the compositions of the capillary basement membrane, we performed multiple labeling of CD31 (PECM1), smooth muscle α-actin (SMA; ACTA2) and collagen IV for endothelial cells, pericytes and basement membrane, respectively. The increased capillary basement membrane thickness in diabetic nerves mainly resulted from the thickening of the basement membrane components (Fig. 1E,F). The presence of cleaved caspase 3(+) endothelial cells provides further signs of microvascular insults in diabetic nerves (Fig. 1G,H).

Given the effects of hyperglycemia and glycation on fibrin structures (Grant, 2007), we next asked whether thrombus formation was enhanced in diabetic nerves and examined its consequences with fibrin immunohistochemistry. All blood vessels and capillaries in control nerves were negative for fibrin (Fig. 1I). By contrast, 16 of 28 (57.1%) sural nerves in the diabetic group had fibrin deposition in both endoneurial capillaries and epineurial vessels (Fig. 1J; Fig. S1), indicating enhanced thrombus formation in diabetic nerves.

**Fig. 2.**
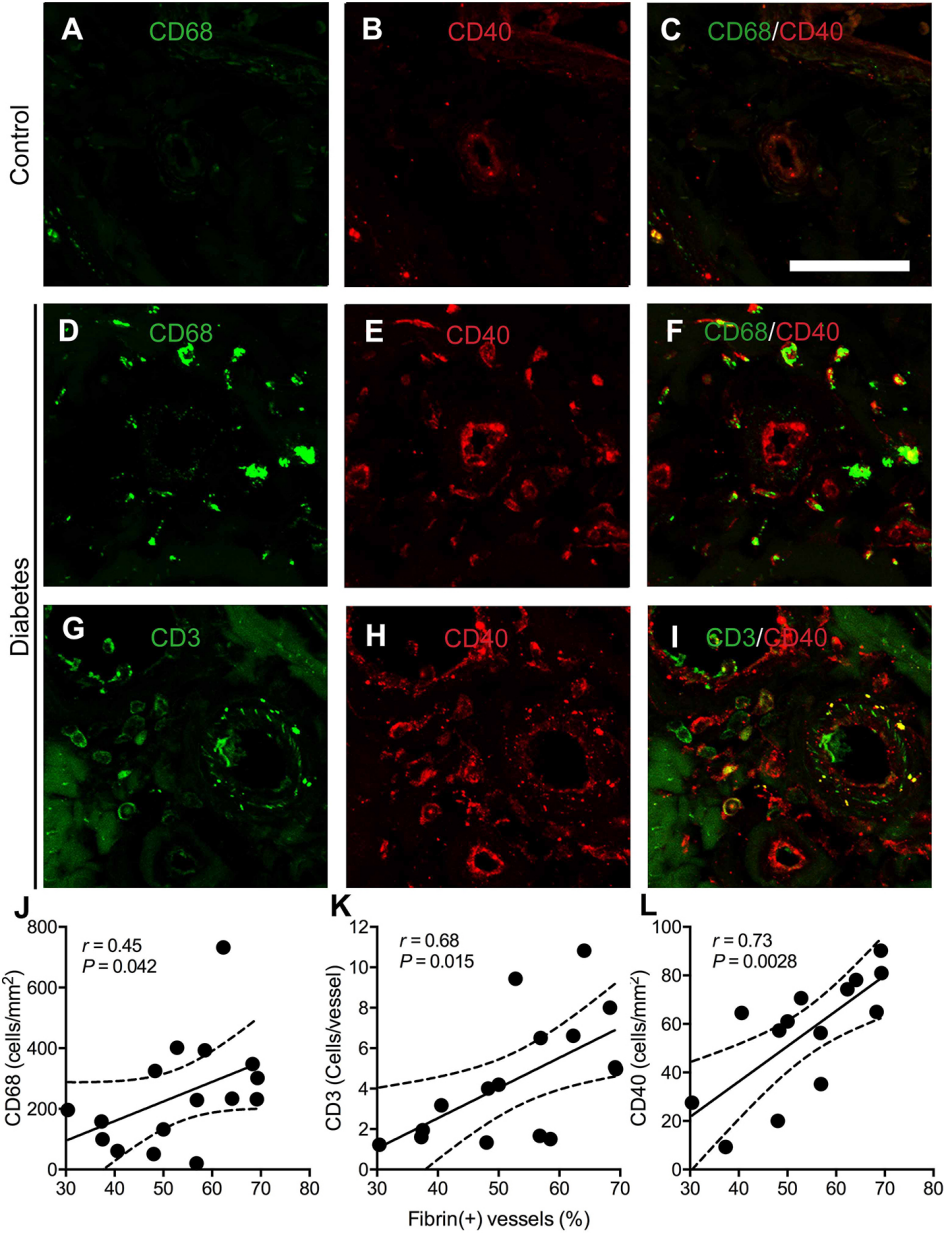
**CD40 expression in endothelial cells and inflammatory cells.** (A-I) Double staining of CD68 (green, A,D), CD3 (green*,* G), and CD40 (red*,* B,E,H) was performed in sural nerves. CD40 immunoreactivity in the endothelial cells of control nerves was minimal but was increased in diabetic nerves. In addition, there was strong colocalization of CD40 with CD68 (F) and CD3 (I) in diabetic nerves. Scale bar: 100 μm. (J-L) In diabetic nerves with fibrin(+) immunostaining (*n*=16), inflammatory cell infiltration and CD40 expression were significantly related to fibrin(+) vessels. The proportions of fibrin(+) vessels are plotted with the infiltration indices of macrophages (J), T cells (K) and CD40(+) cells (L). Solid lines, regression lines; dashed lines, 95% confidence intervals (CIs) by Pearson's correlation analysis.

Because chronic inflammation plays a crucial role in initiating endothelial cell dysfunction and thrombus formation (Libby and Simon, 2001), we first examined the patterns of inflammatory cell infiltration in diabetic nerves, including macrophages (CD68), T cells (CD3 proteins) and B cells (CD20; MS4A1) (Fig. S2). The density of CD68(+) cells was higher in diabetic nerves than in control nerves (244.6±117.0 versus 26.6±19.6 cells/mm^2^, *P*=0.026). T cell infiltration was absent in control nerves, but there was significant infiltration of CD3(+) cells around the epineurial blood vessels of diabetic nerves (4.34±3.04 versus 0±0 cells/vessel, *P*=0.019). CD20 immunoreactivity was absent in the sural nerve of both control and diabetic nerves. Notably, both macrophage and T cell infiltration indices were significantly correlated with the proportion of fibrin(+) blood vessels in diabetic nerves (*r*=0.45, *P*=0.042 for macrophage infiltration index; *r*=0.68, *P*=0.015 for T cell infiltration index; Fig. 2J,K).

Given the enhanced coagulation and inflammation in diabetic nerves, and CD40 has been implicated in the interweaved mechanisms of these complications (Libby and Simon, 2001; Mach et al., 1997), CD40 immunostaining was performed. CD40 immunoreactivity was only minimally detectable in the endothelial cells of control nerves but became upregulated in diabetic nerves (Fig. 2A-I). In diabetic nerves, CD40 expression was enhanced in the endothelial cells and became marked in inflammatory cells. Quantitatively, CD40(+) cells were significantly increased in the endoneurium of diabetic nerves (63.6±29.4 versus 8.9±8.2 cells/mm^2^, *P*=0.002) and were correlated with the proportion of fibrin(+) vasculatures of diabetic nerves (*r*=0.73, *P*=0.0028; Fig. 2L). These results suggested an association of microvascular insults with chronic inflammation, and CD40 might serve as a mediator of enhanced thrombus formation between inflammatory cells and endothelial cells in the diabetic nerves.

### Sural nerve pathology and correlation with microangiopathy

**Fig. 3.**
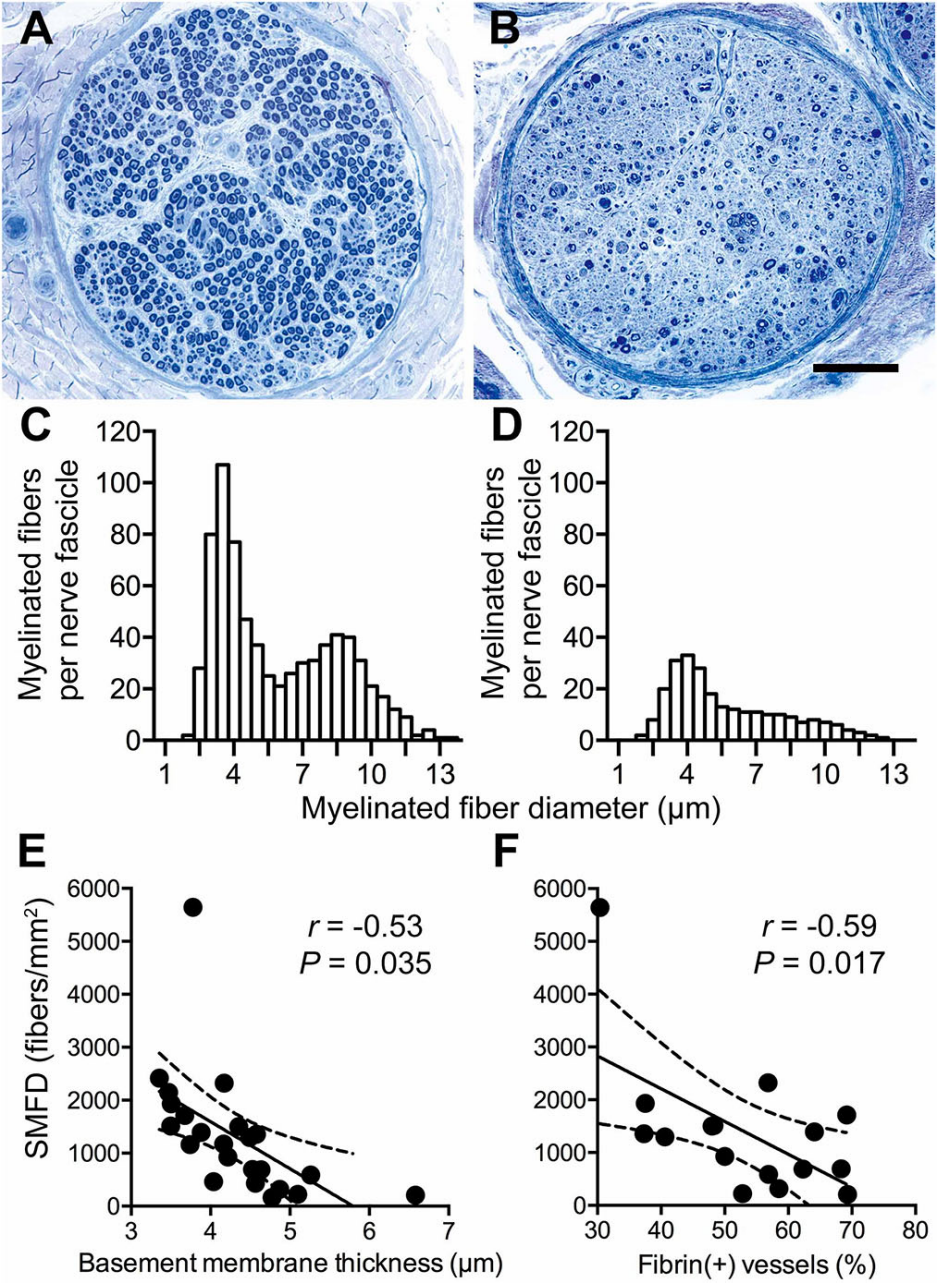
**Nerve degeneration in diabetes.** (A,B) Sural nerves were cut into 1-μm sections and then stained with Toluidine Blue. The sural nerve fascicle from a control subject revealed abundant myelinated fibers (A). By contrast, the diabetic sural nerve fascicle exhibited a marked reduction of both large- and small-diameter myelinated fibers (B). Scale bar: 100 μm. (C,D) The histograms showed the average frequencies of myelinated fibers according to myelinated fiber diameters of the control subjects (C) and diabetes (D). In diabetic nerves (*n=*28), the histogram had a unimodal distribution which was distinct from a bimodal distribution in control nerves (*n=*6) (*χ*^2^=25.7, *P*=0.028 by Chi-squared test). (E,F) SMFD was significantly correlated with capillary basement membrane thickness (*n*=28) and the proportion of fibrin(+) vessels (*n*=16). Solid lines, regression lines; dashed lines, 95% CIs by Pearson's correlation analysis.

To investigate diabetic effects on nerve morphometry, we analyzed nerve pathology and its relationship with microangiopathy. In control subjects, myelinated fibers were abundant with two distinct large and small subpopulations (Fig. 3A). In diabetic nerves, both large and small myelinated fibers were significantly reduced (Fig. 3B). This result was confirmed by the reduced myelinated fiber density (2540±1934 versus 7458±1519 fibers/mm^2^, *P*=0.0001), with a similar reduction in both large (1234±1055 versus 3973±1029 fibers/mm^2^, *P*=0.0002) and small myelinated types (1306±1149 versus 3485±703 fibers/mm^2^, *P*=0.0004). In control nerves, the fiber diameter histogram showed a bimodal distribution, and the pattern became unimodal in diabetic nerves (*P*=0.028 by Chi-squared goodness-of-fit test; Fig. 3C,D). Given this change in the fiber diameter distribution representing patterns of fiber type pathology, we focused on small myelinated fibers in the following analyses.

We further analyzed the relationship between nerve pathology and the parameters of microangiopathy. Capillary basement membrane thickness and the proportion of fibrin(+) blood vessels were determinants of small myelinated fiber density (SMFD) [*r*=−0.53, *P*=0.0035 for capillary basement membrane thickness; *r*=−0.59, *P*=0.017 for the proportion of fibrin(+) blood vessels; Fig. 3E,F]. The above observations indicated that the quantity of small myelinated nerve fibers was correlated with the severity of microangiopathy.

### Expression of HIF-1α, mitogen-activated protein kinase-activated protein kinase 2 and PTEN in sural nerves

**Fig. 4.**
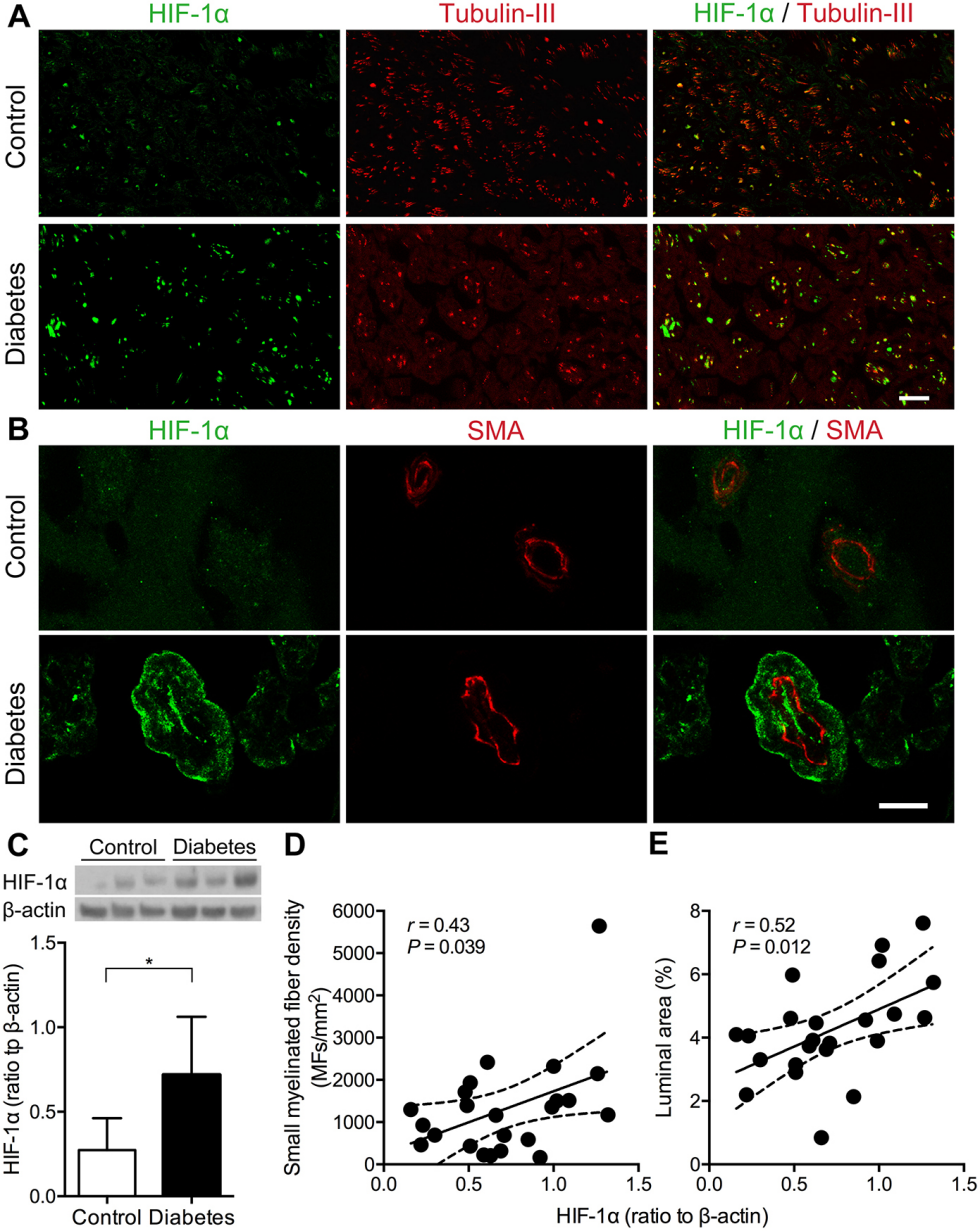
**Upregulation of HIF-1α in diabetic sural nerves.** (A,B) Sural nerve biopsy sections were immunostained. Nerve fibers (A) and endoneurial capillaries (B) were revealed by β-tubulin III and SMA (red), respectively. HIF-1α (green) expression was mainly upregulated in axons, endothelial cells, perivascular infiltrating cells and vascular smooth muscle cells of diabetic patients compared with those of control subjects. Scale bars: (A) 25 μm; (B) 10 μm. (C) HIF-1α expression was validated and quantified with western blotting. The expression of HIF-1α was significantly increased in the sural nerves of diabetic patients (*n*=23) compared with those of control subjects (*n*=3). *P<0.05 by two-tailed Student's *t*-test. (D,E) The HIF-1α expression level of diabetic nerves was plotted against SMFD and capillary luminal area. These parameters were significantly correlated with the HIF-1α expression level among the diabetic group. Solid lines, regression lines; dashed lines, 95% CIs by Pearson's correlation analysis.

**Fig. 5.**
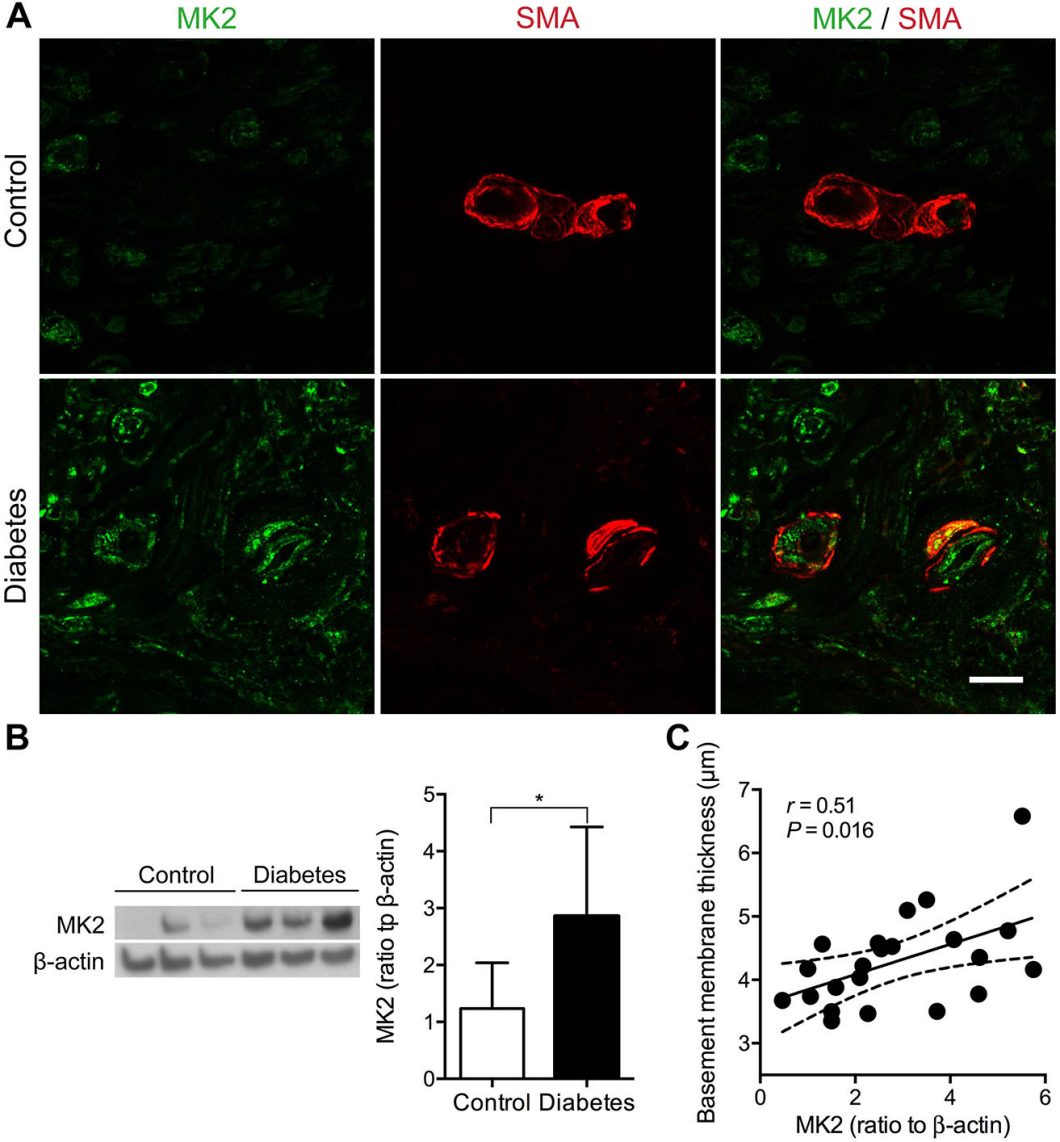
**Upregulation of MK2 in diabetic sural nerves.** (A) Sural nerve biopsy sections were immunostained. Endoneurial capillaries were revealed by SMA (red). MK2 (green) showed minimal expression in the smooth muscle cells and endothelial cells of control subjects, but its expression was increased in the smooth muscle cells and endothelial cells of diabetic patients. Scale bar: 10 μm. (B) The expression of MK2 was quantified with western blotting, and there was a significant increase in MK2 expression in diabetic nerves (*n*=23) compared with control nerves (*n*=3). *P<0.05 by two-tailed Student's *t*-test. (C) The expression of MK2 was significantly correlated with capillary basement membrane thickness among the diabetic group. Solid lines, regression lines; dashed lines, 95% CIs by Pearson's correlation analysis.

**Fig. 6.**
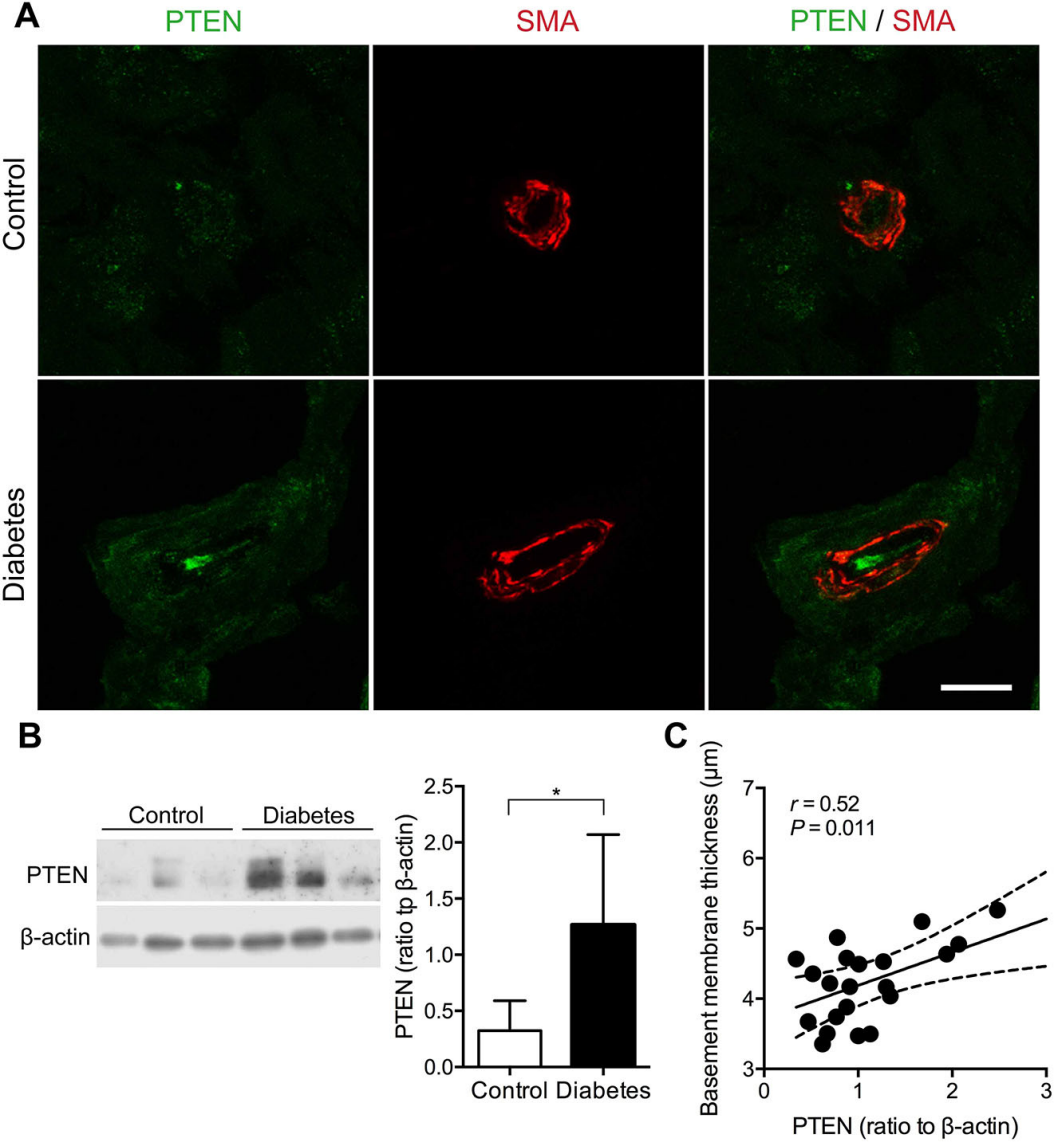
**Upregulation of PTEN in diabetic sural nerves.** (A) Sural nerve biopsy sections were immunostained. Endoneurial capillaries were revealed by SMA (red). PTEN showed limited expression in the endothelial cells of control nerves, but its expression was increased in the smooth muscle cells and endothelial cells of diabetic nerves. Scale bar: 10 μm. (B) Measurement of PTEN expression by western blotting demonstrated that PTEN was significantly increased in diabetic nerves (*n*=23) compared with control nerves (*n*=3). *P<0.05 by two-tailed Student's *t*-test. (C) Capillary basement membrane thickness was significantly correlated with PTEN expression level. Solid lines, regression lines; dashed lines, 95% CIs by Pearson's correlation analysis.

According to the above observations of microangiopathy and its conceivably consequent ischemia, we examined the expression of HIF-1α in sural nerves. HIF-1α immunoreactivity was sparse in control nerves, whereas it was upregulated in the axons and blood vessels of diabetic nerves (Fig. 4A,B). Based on the quantification of western blots, HIF-1α expression was increased in the diabetic patients compared with the controls (0.72±0.34 versus 0.27±0.19, *P*=0.020; Fig. 4C), and was correlated with the SMFD (*r*=0.43, *P*=0.039; Fig. 4D) and capillary luminal area in the diabetic group (*r*=0.52, *P*=0.012; Fig. 4E). Because the MAPK and PI3K (PIK3CA)/Akt signaling pathways regulate HIF-1α (Emerling et al., 2005; Maynard and Ohh, 2007), we examined the expression levels of phosphorylated extracellular signal-regulated kinase 1/2 (pERK1/2), a downstream molecule of the MAPK/ERK pathway, mitogen-activated protein kinase-activated protein kinase 2 (MK2; MAPKAPK2), a key enzyme marker of the p38 MAPK pathway, and PTEN, an inhibitory molecule of the PI3K/Akt pathway. pERK1/2 immunoreactivities were similar in both control and diabetic sural nerves (Fig. S3). MK2 immunoreactivity was minimal in control nerves but became abundantly expressed in the microvasculature of diabetic nerves (Fig. 5A; Fig. S4A). For quantitative comparisons, MK2 expression was increased in diabetic nerves compared with control nerves (2.86±1.57 versus 1.24±0.80, *P*=0.047; Fig. 5B), and was correlated with the capillary basement membrane thickness in the diabetic group (*r*=0.51, *P*=0.016; Fig. 5C). In control nerves, there was limited PTEN immunoreactivity. By contrast, PTEN immunoreactivity was increased in the blood vessels of diabetic nerves (Fig. 6A; Fig. S4B). Quantitatively, PTEN expression was increased in diabetic nerves compared with control nerves (1.27±0.80 versus 0.32±0.27, *P*=0.030; Fig. 6B), and was correlated with the capillary basement membrane thickness in the diabetic group (*r*=0.52, *P*=0.011; Fig. 6C). Taken together, HIF-1α, MK2 and PTEN expression all correlated with the morphometric index of vascular integrity, i.e. capillary luminal area or capillary basement membrane thickness, indicating that the activation of these molecules was associated with diabetes-induced microvascular insults.

### Molecular alterations in human umbilical vein endothelial cell cultures exposed to high-glucose medium

To investigate whether the above molecular alterations were casually related to hyperglycemia, we examined the molecular expression levels in human umbilical vein endothelial cells (HUVECs) cultured in high-glucose medium. After exposure to added 3, 15 and 30 mM D-glucose for 7 days, there was significant dose-dependent upregulation of HIF-1α (*P*=0.038), MK2 (*P*=0.034), PTEN (*P*=0.033) and CD40 (*P*=0.031), particularly in the HUVEC cultures exposed to added 30 mM D-glucose (Fig. S5). These *in vitro* findings recapitulated those in the endothelial cells in diabetic nerves, indicating the detrimental effects of a high-glucose environment on endothelial cells, resulting in alterations of molecular signatures.

### CD40 regulates HIF-1α and PTEN, but not MK2, in HUVECs

**Fig. 7.**
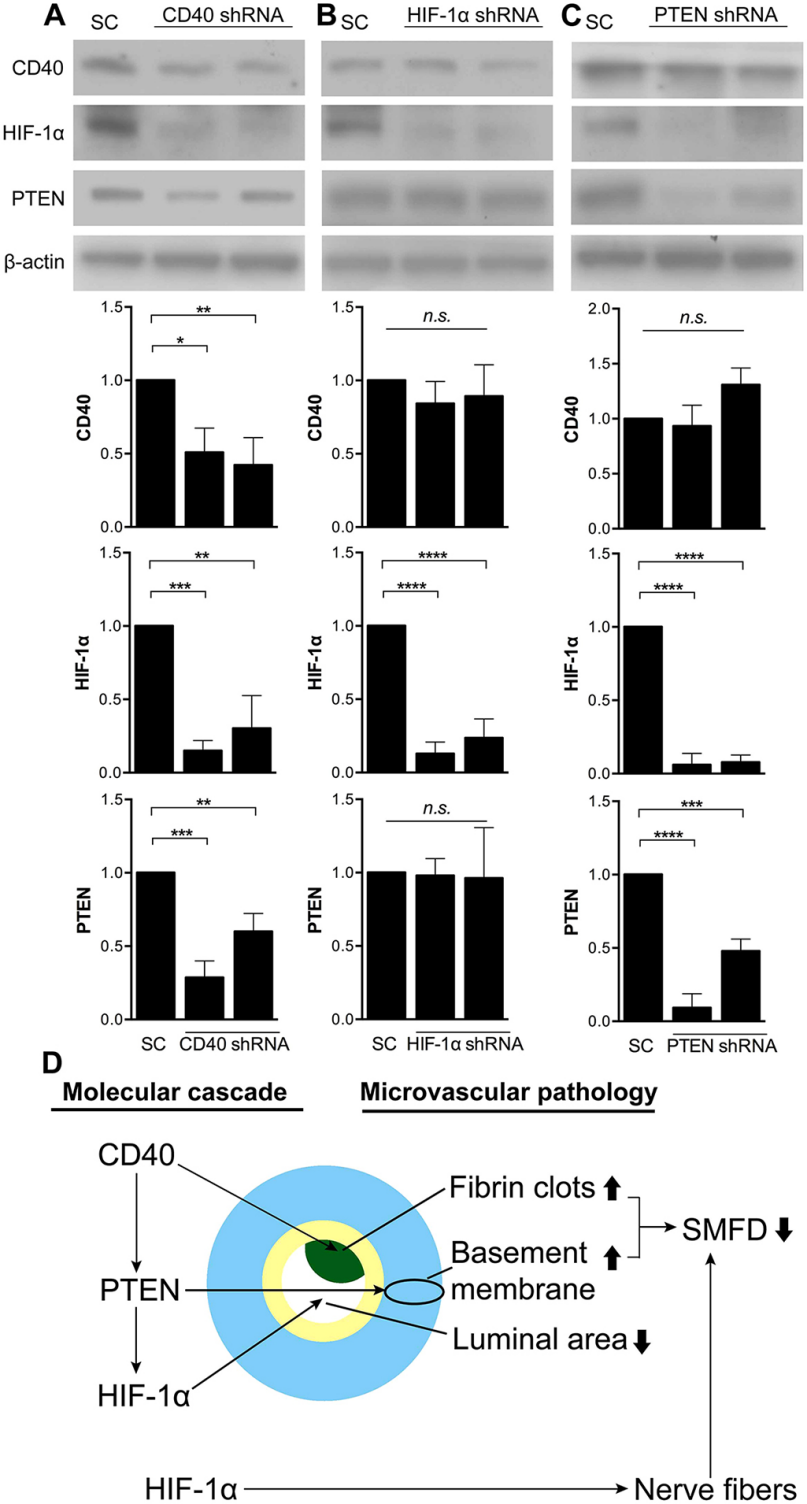
**CD40 knockdown and putative signaling pathway in HUVECs after exposure to high-glucose medium.** (A-C) HUVECs were infected with CD40 shRNA, HIF-1α shRNA and PTEN shRNA. Following lentiviral infection for 24 h, HUVECs were exposed to high-glucose (30 mM D-glucose) medium for 7 days. Western blotting using anti-CD40, anti-HIF-1α and anti-PTEN antibodies confirmed the knockdown efficiency of the molecules. CD40 knockdown was associated with downregulation of HIF-1α and PTEN (A), whereas the expression of CD40 remained unchanged after knockdown of HIF-1α or PTEN (B,C). Knockdown of HIF-1α did not affect the expression of PTEN (B). By contrast, knockdown of PTEN significantly reduced the expression of HIF-1α (C). The mean results of three independent experiments (±s.d.) are shown, and data were normalized using the amount of protein expression of a scrambled construct (SC). n.s., not significant. *P<0.05, **P<0.005, ***P<0.0005, ****P<0.0001 by one-way ANOVA with Dunnett's post hoc test. (D) The knockdown experiments demonstrated the hierarchy of the high-glucose-induced signaling pathway. The illustration summarizes the associations between the molecular signatures and morphometry in the capillaries of diabetic sural nerves.

Given that CD40 induces intracellular signaling in endothelial cells through the PI3K/Akt and p38 MAPK pathways (Deregibus et al., 2003; Chakrabarti et al., 2007), we next silenced CD40 in HUVECs with lentivirus-based short hairpin RNA (shRNA) and then cultured the cells in a medium containing added 30 mM D-glucose for 7 days to examine the effects on molecular cascades of microangiopathy. The knockdown effects among the four constructs were assessed with western blotting. Two shRNAs with higher knockdown efficiency were used in the subsequent experiments. CD40 shRNA significantly silenced CD40 expression in HUVECs (*P*=0.0054 by one-way ANOVA; Fig. 7A). Accordingly, HIF-1α and PTEN were significantly downregulated in concert with CD40 by CD40 shRNA (*P*=0.0005 by one-way ANOVA for HIF-1α; *P*=0.0003 by one-way ANOVA for PTEN). However, there was no difference in MK2 expression following CD40 silencing (Fig. S6), indicating that HIF-1α and PTEN were downstream molecules of the CD40 signaling pathway.

To clarify the relationship between CD40, HIF-1α and PTEN, we performed knockdown experiments on HIF-1α (Fig. 7B) and PTEN (Fig. 7C). Western blotting demonstrated that the silencing of HIF-1α or PTEN had no effect on CD40 expression. By contrast, inhibition of PTEN resulted in HIF-1α downregulation (*P*<0.0001 by one-way ANOVA), while PTEN expression was similar following HIF-1α knockdown. Taken together, these above observations proposed a hierarchy of molecular signaling pathways from CD40 to HIF-1α (Fig. 7D).

## DISCUSSION

This report documented CD40 as the major molecular signature of microangiopathy in diabetic nerve pathology from the dimensions of inflammation-related thrombosis. We demonstrated (1) microangiopathy (basement membrane thickness and thrombosis) as a determinant of small myelinated fiber degeneration, (2) increased inflammation with a pro-coagulation cascade, (3) the altered expression levels of HIF-1α, MK2 and PTEN and their correlations with the pathologic severity of diabetic microvasculature (Fig. 7D), and (4) direct evidence of CD40 upregulation induced by high-glucose exposure resulting in the expression of HIF-1α and PTEN *in vitro*.

In diabetic patients, vascular integrity (the thickness and patency of endoneurial capillaries) was a significant determinant of nerve degeneration, far more important than the quantity of endoneurial capillaries, i.e. endoneurial capillary density. Despite the similar quantity of endoneurial capillaries between diabetic nerves and control nerves, the quality of capillaries in diabetic nerves was much worse than that of control nerves, as assessed by capillary basement membrane thickness, capillary luminal area and fibrin clots in blood vessels, recapitulating the vasculopathy in ischemic limbs caused by peripheral vascular disease. However, the global and generalized loss of nerve fibers in the current study was distinct to focal nerve fiber injury involving certain fascicles or fibers in peripheral vascular disease (Nukada et al., 1996).

This study provided the morphometry evidence of microvascular pathology, i.e. basement membrane thickness and fibrin(+) clots in capillaries as the major contributors of SMFD, which was in contrast to most studies considering myelinated fibers as a whole. Despite the fact that both large and small myelinated fiber densities were diminished in diabetic patients, small nerve fibers are more vulnerable to diabetic insults, and nerve regeneration is frequently observed in diabetic patients (Løseth et al., 2008), indicating that the role of small myelinated fibers is much more exclusive than that of large myelinated fibers. In addition, given that small myelinated fibers contribute to skin nerve terminals (Herrmann et al., 1999) and small fiber neuropathy constitutes an integral part of diabetic neuropathy (Shun et al., 2004), it is important to determine how small myelinated fibers were affected in diabetes-induced nerve degeneration.

This study provided direct evidence for the simultaneous existence of two pathological processes, inflammation and thrombosis, in microangiopathy of diabetic neuropathy. Although inflammation has been proposed as an important pathophysiology associated with diabetes, direct evidence of inflammation as an important pathology in diabetic neuropathy is sparse. The current report documented that diabetic microvasculature was associated with the inflammation of macrophage and T cell infiltration. Most macrophages expressing CD68, a total infiltrated macrophage marker (Wentworth et al., 2010), were also immunoreactive for CD40 as one of the M1 polarization markers (Vogel et al., 2014), indicating that the inflammatory response was prompted in diabetic sural nerves. Furthermore, the presence of fibrin(+) clots in diabetic microangiopathy indicated that the inflammatory response resulting from long-term diabetes could serve as an important mechanism for thrombus formation. A critical question was whether there was a molecular candidate contributing to both pathological processes of inflammation and thrombosis. The upregulation of CD40 in endothelial cells, macrophages and T cells in diabetes suggested that enhanced CD40 expression could play a key role in mediating leukocyte adhesion through the CD40-CD40L signaling pathway, resulting in the induction of pro-inflammatory and pro-thrombotic processes (Libby and Simon, 2001; Lutgens et al., 2007). Taken together, the association of CD40 with the proportion of thrombosed capillaries in diabetic neuropathy underpinned the interactions between these two networks of inflammation and thrombosis in the pathogenesis of microangiopathy leading to nerve degeneration.

This study demonstrated the three key molecular signatures of microangiopathy-mediated diabetic neuropathy downstream of CD40: HIF-1α, MK2 and PTEN. According to previous studies, the functions of HIF-1α are controversial. For example, HIF-1α induces erythropoietin to promote neuron survival (Ostrowski et al., 2011), but another study indicated that HIF-1α promotes cell death in the context of cerebral ischemia via p53 (Halterman et al., 1999). Although diabetes might have a complex repressive effect on the stabilization and transactivation of HIF-1α (Bento and Pereira, 2011; Catrina et al., 2004), our results indicated that HIF-1α expression was associated with better nerve profiles. In diabetes, this correlation presumably resulted from stimulating the recovery of blood flow, similar to the induction of HIF-1α in the ischemic limb (Sarkar et al., 2009). This assumption was comparable to our findings, i.e. HIF-1α was also positively associated with the luminal area of microvessels. Whether microangiopathy coexisted with severe diabetic neuropathy or was the etiology of nerve degeneration has been an issue of debate for decades. The current study suggested that tissue hypoxia might occur prior to nerve degeneration and that the capability for expressing HIF-1α was related to the protection of nerve integrity. Further investigation on a cohort study of diabetic patients could clarify this assumption.

In experimental diabetes, the p38 MAPK and PI3K/Akt signaling pathways have been thought to be responsible for reduced nerve conduction velocity and the impaired nerve regeneration of diabetic axons, respectively (Price et al., 2004; Singh et al., 2014). This study identified additional mediators of MK2 and PTEN in disrupted molecular cascades in epineurial blood vessels and endoneurial capillaries, i.e. the upregulation of both MK2 and PTEN was associated with the degree of microangiopathy. Given that the p38 MAPK and PI3K/Akt pathways are involved in the regulation of cell survival, this observation might indicate that pericytes, smooth muscle cells and endothelial cells undergo apoptosis through the activation of the p38 MAPK pathway and inhibition of the PI3K/Akt pathway. These molecular alterations underlie the mechanisms of thickened capillary basement membrane in diabetic neuropathy, which included the cell debris of smooth muscle cells and pericytes (Giannini and Dyck, 1994). In addition, MK2 is crucial for collagen IV degradation by regulating the expression of matrix metalloproteinase-2 (MMP-2) (Xu et al., 2006), possibly influencing the inflammatory infiltration of macrophages and T cells. A seminal study identified a set of gene signatures that classified the progression of diabetic neuropathies (Hur et al., 2011). The current study thus provides another dimension to classify diabetic neuropathies by molecular signatures.

In addition to thickened basement membrane, diabetes contributes to inflammation and coagulation in the microvasculature (Mach et al., 1997; Libby and Simon, 2001). CD40 plays an intermediary role in these diabetic complications. By culturing HUVECs with high-glucose medium as a model mimicking microangiopathy in uncontrolled hyperglycemia of diabetes (Cagliero et al., 1991; Rubenstein et al., 2011; Patel et al., 2013), we demonstrated, for the first time, that CD40 was induced by high-glucose exposure. We chose to investigate the relationship between HIF-1α, MK2 and PTEN, and CD40 as they were upregulated in the endothelial cells of sural nerve microvasculatures and were all major determinants of microangiopathy and nerve morphometry in diabetic neuropathy. Although the PI3K/Akt and p-38 MAPK signaling pathways are involved downstream of CD40 (Deregibus et al., 2003; Chakrabarti et al., 2007; Dormond et al., 2008), our data suggested that CD40 initiated the high-glucose-induced upregulation of PTEN, indicating suppressed PI3K/Akt signaling. Therefore, an increase in MK2 under the high-glucose condition possibly resulted from hypoxia and the inflammatory response (Limbourg et al., 2015). Although it was not clear which process was the most major one in HIF-1α accumulation induced by high-glucose exposure (Yan et al., 2012), we provided direct evidence that the regulation of HIF-1α was, in part, associated with PTEN as the intermediate molecule in the CD40 signaling pathway. Collectively, these observations support the notion that metabolic derangements resulting from hyperglycemia induced by diabetes disrupt the molecular profiles in endothelial cells. Furthermore, the current study provides a putative signaling pathway that could be a therapeutic target for diabetic complications; that is, because HIF-1α was beneficial for the integrity of the sural nerve, a combination of HIF-1α overexpression and CD40 inhibition could be a therapeutic strategy for improving microvascular and nerve integrity in diabetic neuropathy.

### Conclusion

In summary, this study demonstrated that microangiopathy, including morphological and molecular alterations, is a major pathology substrate of diabetic nerve degeneration, and provides evidence that the CD40 signaling pathway mediates the interactions between inflammation and thrombosis in diabetes. Furthermore, knockdown of CD40 attenuates high-glucose-induced upregulation of HIF-1α and PTEN *in vitro*. However, this cross-sectional study might be limited by the fact that the sural nerves came from an advanced stage of neuropathy with vasculopathy as the primary etiology. Ideally, diabetic microangiopathy without neuropathy would be the best control. Nevertheless, this report provides new insights into microangiopathy and its contribution to nerve pathology. Certainly, further studies are mandatory to include diabetic microangiopathy in the absence of neuropathy for a prospective design.

## MATERIALS AND METHODS

### Study subjects, demographic data and nerve morphometry

This study examined the sural nerves from type 2 diabetic patients from National Taiwan University Hospital (Taipei, Taiwan). Patients usually visited the hospital once every 1-3 months for adjustments of diabetic control. All glycemic data were collected for analysis before the nerve biopsy. Control sural nerves came from a portion of the sural nerve during grafting for subjects with nerve trauma. Sural nerve biopsies were obtained from a standard site posterior to the lateral malleolus under local anesthesia or from the same site of amputated limbs. The study was approved by the Institutional Review Board at National Taiwan University Hospital, and signed written consent forms were obtained from all participants in order to use the biopsies and recordings during their treatments. All clinical investigation was conducted according to the principles expressed in the Declaration of Helsinki.

There were 28 type 2 diabetic patients (15 males and 13 females). Among these patients, five had sural nerves taken for regular diagnostic biopsy, and 23 had sural nerves taken at the time of amputation (Table 1). All amputated patients could ambulate independently before amputation. There were six healthy control subjects (two males and four females, aged 44.6±25.5 years) who were determined to be neuropathy-free by neurological examination and symptom score. There was no difference in age between the biopsy subgroup of diabetic patients and control subjects (adjusted *P*=0.943 by one-way ANOVA with Tukey's post hoc test). Control subjects and the biopsy subgroup of diabetic patients were younger than the amputated subgroup of diabetic patients (adjusted *P*=0.008 and *P*=0.021, respectively, by one-way ANOVA with Tukey's post hoc test). Because age was a potential confounder of microangiopathy, we addressed this issue with multiple linear regression models (Table S1), with each morphometric parameter as a dependent variable, and age and disease status (control versus diabetes) as independent variables. Disease status, i.e. diabetes, was associated with each morphometric parameter, indicating that the effects of diabetes on microangiopathy were independent of age. There was no difference in plasma glucose, nerve fiber densities, and microvascular morphometry between the two subgroups of diabetic patients (Table 1). Thus, the data of these two subgroups were pooled together in the following analyses and presentations.

**Table 1.**
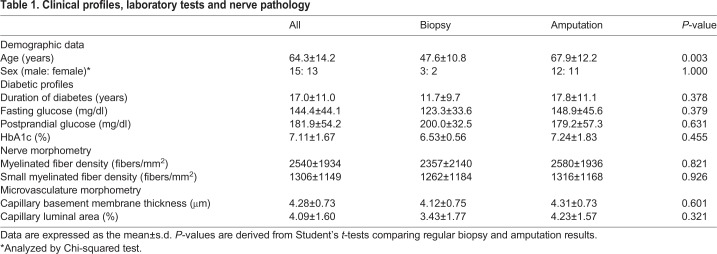
**Clinical profiles, laboratory tests and nerve pathology**

### Antibodies

Anti-CD31 (1:500, M0834), anti-CD68 (1:50, M0876), anti-CD3 (1:50, M0742), anti-CD20 (1:50, CD20cy), and fluorescein isothiocyanate (FITC)-conjugated anti-fibrin (1:100, F0111) primary antibodies were purchased from Dako (Glostrup, Denmark); anti-SMA (1:1000, A5228) and β-tubulin III (1:1000, T5076) were obtained from Sigma-Aldrich (St. Louis, MO, USA); anti-collagen IV (1:50, 2150-0140) and anti-CD40 (1:100, MCA 1590) were purchased from AbD Serotec (Oxford, UK); anti-pERK1/2 (1:100, #9101) and anti-PTEN (1:500, #9559) were purchased from Cell Signaling Technology (Danvers, MA, USA); anti-HIF-1α (1:100, sc-10790) was obtained from Santa Cruz Biotechnology (Santa Cruz, CA, USA); anti-MK2 (1:200, ADI-KAP-MA015) was obtained from Stressgen (Victoria, Canada). Alexa Fluor 488-conjugated goat anti-rabbit IgG, Alexa Fluor 488-conjugated goat anti-mouse IgG1a, Cy3-conjugated goat anti-mouse IgG2a and Alexa Fluor 647-conjugated donkey anti-rabbit IgG secondary antibodies were purchased from Jackson ImmunoResearch Laboratories (West Grove, PA, USA).

### Morphometry of nerve and microvascular pathologies

Glutaraldehyde-fixed nerves were post-fixed in 2% osmium tetroxide for 2 h, dehydrated through a graded ethanol series, and embedded in Epon 812 resin (Polyscience, Philadelphia, PA, USA). Semithin sections were cut on a Reichert Ultracut E (Leica, Wetzler, Germany) and stained with Toluidine Blue.

Myelinated fibers were photographed at an original magnification of ×20, and endoneurial capillaries in each fascicle were photographed at an original magnification of ×100 under a Leica DM2500 microscope. Morphometric parameters were measured using Image-Pro PLUS software (Media Cybernetics, Silver Spring, MD, USA). All myelinated fibers in the entire fascicle were counted and were expressed as fibers/mm^2^ and further classified as large and small myelinated fibers, with 5 μm as the cutoff (Thomas et al., 2001). The capillary pathology criteria were based on previous studies (Dyck et al., 1985; Estrella et al., 2008). Briefly, the capillary basement membrane thickness was assessed by the mean width from the base of endothelial cells to the outline of the basement membrane of pericytes; and the luminal area was depicted by the outline of endothelial cells facing the lumen using Image-Pro PLUS software. Endoneurial capillaries in each endoneurium of all nerve fascicles were counted and divided into each comparative area of endoneurium. The endoneurial capillary density was derived from the average of each nerve fascicle and was expressed as capillaries/mm^2^.

### Immunofluorescence and quantification of microvascular insults

Sural nerve specimens were fixed overnight in 2% paraformaldehyde-lysine-periodate and then changed to phosphate buffer for storage. Frozen sections (10 μm) were immunostained following established protocols (Hsieh et al., 2008). Briefly, nonspecific binding was blocked by 0.1% Triton X-100 and 0.5% nonfat milk in Tris buffer, and nerve sections were incubated at 4°C overnight with primary antibodies suspended in 0.1% Triton X-100 and 0.5% nonfat milk in Tris buffer. After the sections were rinsed with Tris buffer, they were incubated with secondary antibodies at room temperature for 1 h. After a rinse in Tris buffer, 4′,6-diamidino-2-phenylindole (DAPI, Sigma-Aldrich) was used for nuclear staining, if necessary. Fluorescent samples were viewed and scanned under a Leica SP5 confocal imaging system.

For fibrin quantification, the proportion of fibrin(+) blood vessels was derived according to the number of fibrin(+) blood vessels divided by the total number of blood vessels in that nerve tissue. For CD40 quantification, CD40(+) cells in the endoneurium of all nerve fascicles were counted and divided by the area of endoneurium and were expressed as cells/mm^2^.

### Immunohistochemistry and quantification of inflammatory cell infiltration

Additional sural nerve frozen sections were quenched by 1% H_2_O_2_ and were immunohistochemically stained at 4°C overnight for CD68, CD3 and CD20 with mouse spleen as positive controls. Sections were incubated with biotinylated goat anti-rabbit IgG (Vector Laboratories, Burlingame, CA, USA) at room temperature for 1 h, followed by incubation with the avidin-biotin complex (Vector Laboratories) for 1 h. Diaminobenzidine (DAB, Sigma-Aldrich) was used as the chromogen.

For macrophage quantification, CD68(+) cells in each endoneurium of all nerve fascicles were counted and divided by each comparative area of endoneurium. For T cell quantification, CD3(+) cells around epineurial blood vessels (within 100 μm) were counted and divided by the number of epineurial blood vessels. The macrophage infiltration and T cell infiltration indices were expressed as cells/mm^2^ and cells/vessel, respectively.

### HUVEC culture and high-glucose exposure

HUVECs were purchased from Bioresource Collection and Research Center (Hsinchu, Taiwan). Cultures were maintained with M199 (Sigma-Aldrich) containing 10% fetal bovine serum (Gibco, Grand Island, NY, USA), 30 μg/ml endothelial cell growth supplement (Merck Millipore, Darmstadt, Germany), 25 U/ml heparin (Sigma-Aldrich), 100 units/ml penicillin (Gibco), and 100 μg/ml streptomycin (Gibco) at 37°C with 5% CO_2_/95% air in a humidified environment; and the entire medium was replenished every 2-3 days. HUVECs at passage 4-8 were used in the experiments. For high-glucose exposure, D-glucose stock solution was added to the growth medium to achieve the final added concentrations of 3, 15 and 30 mM. The cell medium was changed, and high glucose was freshly added every other day for 7 days.

For protein knockdown, lentiviruses expressing shRNA targeting CD40, HIF-1α and PTEN were produced and designed by RNAi Core, Academia Sinica, Taiwan (Table S2). The PLKO.1 expression vector was used for shRNA expression. HUVECs (seeded at 2800 cells/mm^2^ cell density) were infected with the indicated lentivirus (multiplicity of infection=3) containing 8 μg/ml polybrene for 24 h and with fresh medium containing 1 μg/ml puromycin and incubated for 48 h to select infected cells. HUVECs were further exposed to added 30 mM D-glucose for 7 days and were then lysed in radioimmunoprecipitation assay (RIPA) buffer with a cocktail of protease inhibitors (Sigma-Aldrich) for analysis of proteins using western blotting.

### Western blot analysis

For each sural nerve, a 0.5-cm segment was desheathed by removing most of the connective tissue in the epineurium and homogenized with ice-cold lysis buffer containing proteinase inhibitor cocktail (Sigma-Aldrich). Homogenates were centrifuged at 12,000 ***g*** at 4°C for 15 min, and the supernatants were collected. Proteins (10 μg) were separated by 10% (w/v) SDS-PAGE and were transferred to immobilon polyvinylidene difluoride membranes (Millipore, Billerica, MA, USA). Nonspecific binding sites were blocked with 5% (w/v) nonfat milk for 1 h, and blots were incubated with anti-HIF-1α, anti-MK2 or anti-PTEN antibody suspended in 5% nonfat milk at 4°C overnight. After washing in buffer (0.05 M Trizma base, 0.5 M NaCl and 0.5% Tween 20), the blots were incubated with horseradish peroxidase-linked secondary antibodies (Promega, Madison, WI, USA) at room temperature for 1 h. The blots were washed again, and the bands were detected using enhanced chemiluminescence (Thermo Fisher Scientific, Rockford, IL, USA) and were exposed to medical X-ray film (Fuji, Tokyo, Japan). The relative density of protein bands was analyzed by ImageJ v.1.47 h (National Institutes of Health, MD, USA), and the relative density of each protein band was normalized to the corresponding amount of β-actin.

### Statistical analysis

Data were analyzed by statistical software Prism 6 (GraphPad Software, San Diego, CA, USA). Numeric variables are expressed as the mean±s.d. and were compared using two-tailed Student's *t*-tests if the data followed a Gaussian distribution. The differences in age between control subjects, the biopsy subgroup of diabetic patients and the amputated subgroup of diabetic patients were analyzed by one-way ANOVA with Tukey's post hoc test. The comparisons of sex and the distribution of nerve fiber diameter were analyzed by Chi-squared goodness-of-fit test. Pearson's correlation between variables was analyzed with the slope of the regression line, including the 95% confidence interval (CI). One-way ANOVA with Dunnett's post hoc test was used to evaluate the effects of different shRNA constructs on protein expression. The results were considered significant at *P*<0.05.

## Supplementary Material

Supplementary information
