# Supplementary material for: CD40-mediated HIF-1α expression underlying microangiopathy in diabetic nerve pathology
Source: Dis Model Mech. 2018 Apr 26;11(4):dmm033647. doi: 10.1242/dmm.033647 (PMC5963861; doi:10.1242/dmm.033647)
Supplement: Supplementary information [file dmm-11-033647-s1.pdf]

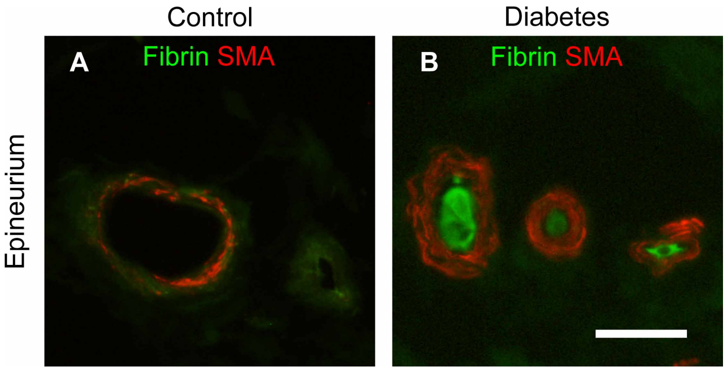

Fig. S1. Thrombosis in diabetic blood vessels.

Thromboses containing fibrin clots were stained with FITC-conjugated fibrin antibody (green), and epineurial blood vessels were revealed by SMA (red) immunohistochemistry. In the epineurium of sural nerves, fibrin(+) clots were absent and SMA staining was minimal in control blood vessels (**A**), whereas there were fibrin(+) clots with markedly increased SMA staining (**B**) in diabetic blood vessels. Scale bars: 50 µm.

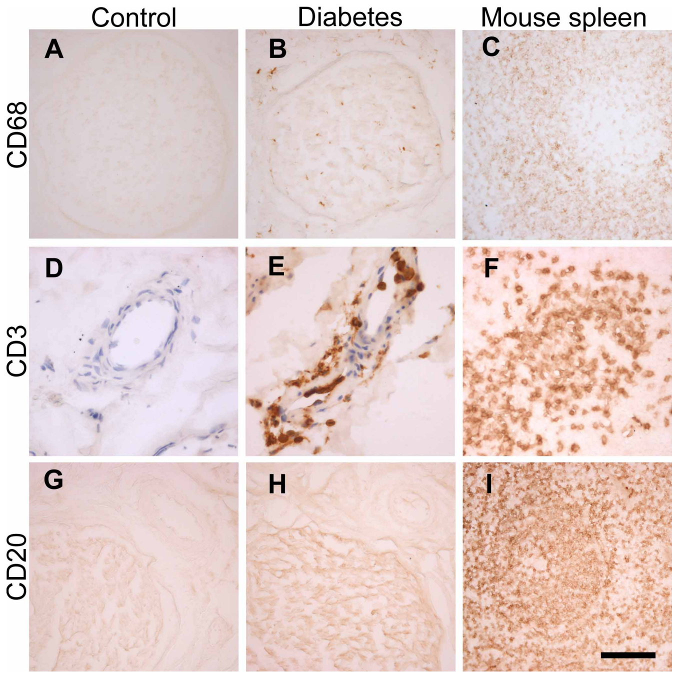

Fig. S2. Infiltration of inflammatory cells in diabetic nerves.

Inflammatory infiltration of macrophages, T cells, and B cells was investigated with CD68 (**A**-**C**), CD3 (**D**-**F**), and CD20 (**G**-**I**) immunostaining, respectively. (**A**,**D**,**G)** No inflammatory cells were noted in control nerves. (**B**,**E**,**H**) In diabetic nerves, macrophage infiltration was detectable in both the epineurium and endoneurium. T cells were mainly located near epineurial blood vessels. No B cells were detected in diabetic nerves. (**C**,**F**,**I**) Mouse spleen was used as a positive control. Scale bar: 100 µm (**A**-**C** and **G**-**I**); 50 µm (**D**-**F**).

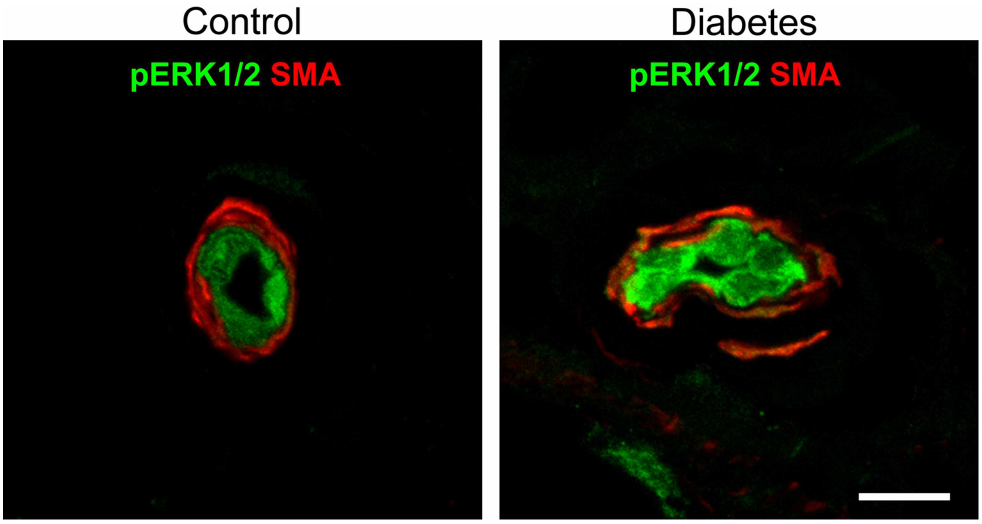

Fig. S3. Similar pERK1/2 expression in control and diabetic nerves.

Endoneurial capillaries were revealed by SMA (red). pERK1/2 (green) was mainly detected in the endothelial cell of endoneurial blood vessels, which showed similar expression patterns between control and diabetic nerves. Scale bar: 10 µm.

Disease Models & Mechanisms • Supplementary information

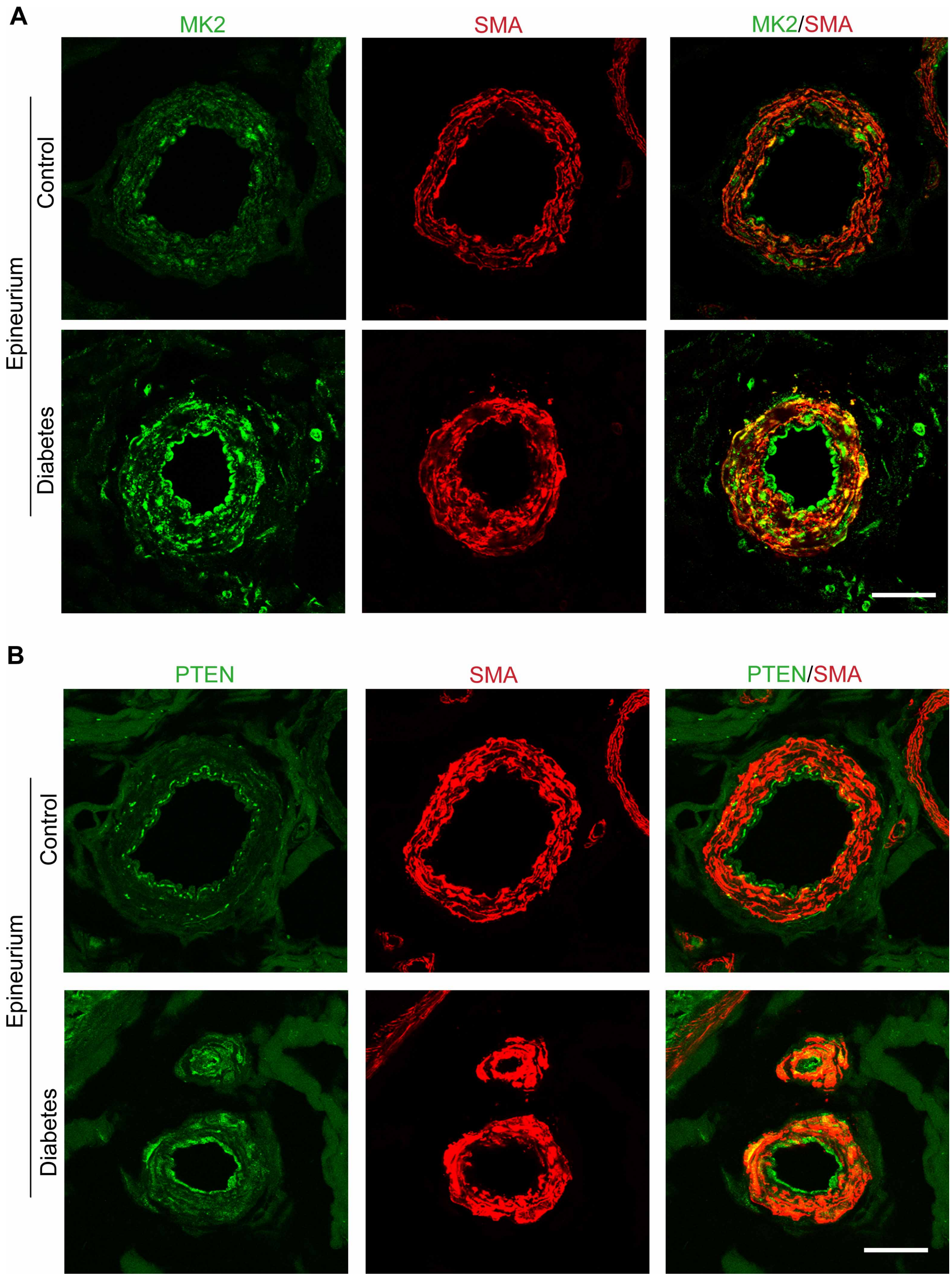

Fig. S4. Upregulation of MK2 and PTEN in diabetic sural nerves.

Sural nerve biopsy sections were immunostained. Epineurial blood vessels were revealed by SMA (red). MK2 (green, **A**) and PTEN (green, **B**) showed minimal expression in the smooth muscle cells and endothelial cells of control subjects, but the expression was increased in the smooth muscle cells and endothelial cells of diabetic patients. Scale bars: 25 μm.

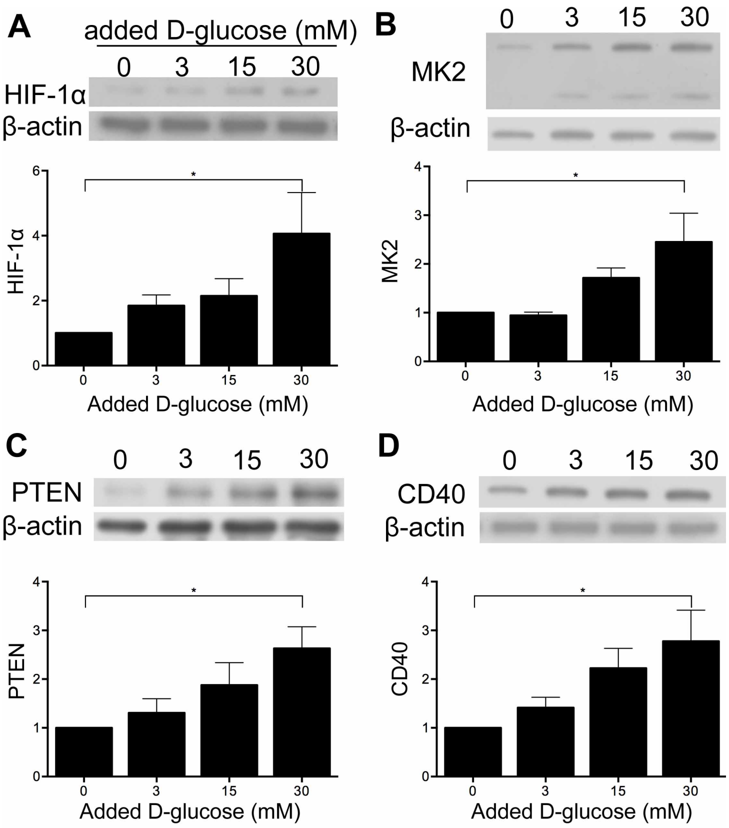

Fig. S5. Altered molecular signatures in HUVEC cultures treated with high-glucose medium.

HUVEC cultures were treated with final added D-glucose concentrations of 3, 15, and 30 mM in the complete medium for 7 days. Western blots showed upregulation of HIF-1α (**A**), MK2 (**B**), PTEN (**C**), and CD40 (**D**) in a dose-dependent manner. The mean results of three independent experiments (± s.d.) are shown, and the data were normalized using the amount of protein expression without added glucose. *$P < 0.05$ by 1-way ANOVA with Dunnett's post hoc test.

Disease Models & Mechanisms • Supplementary information

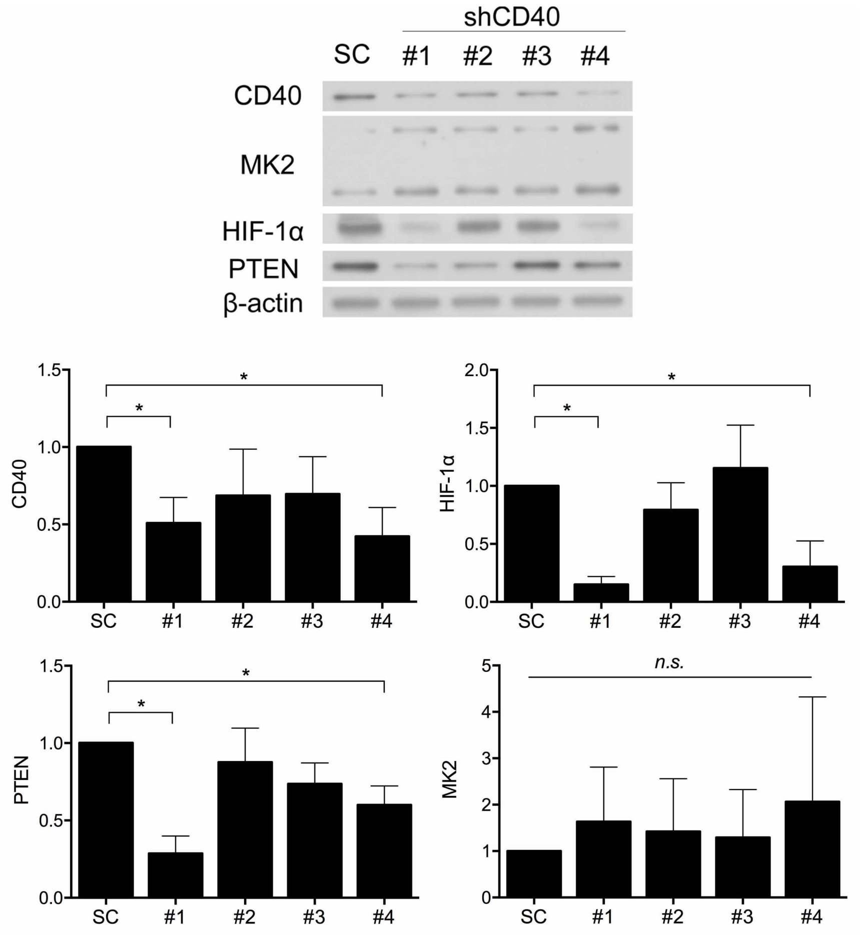

Fig. S6. Western blots of molecular expression by silencing CD40 in HUVECs.

HUVECs were infected with CD40 shRNA (shCD40). Following lentiviral infection for 24 h, HUVECs were exposed to high-glucose (30 mM D-glucose) medium for 7 days. Western blot using anti-CD40, anti-MK2, anti-HIF-1α, and anti-PTEN antibodies confirmed the knockdown efficiency of CD40 and showed down-regulation of HIF-1α and PTEN, while the expression of MK2 remained unchanged by the knockdown of CD40. The mean results of three independent experiments (± s.d.) are shown, and the data were normalized using the amount of protein expression of a scrambled construct (SC). n.s. = not significant. *$P < 0.05$ by 1-way ANOVA with Dunnett's post hoc test.

Disease Models & Mechanisms • Supplementary information

Table S1. Multiple linear regression models

	Model Adjusted R^2, *P*	Age *t*, *P*	Disease‡ (control vs. diabetes) *t*, *P*
Nerve morphometry			
Myelinated fiber density	0.56, < 0.0001	2.74, 0.010	-6.45, <0.0001
Small myelinated fiber density	0.42, 0.0001	2.44, 0.021	-4.91, < 0.0001
Microvasculature morphometry			
Capillary basement membrane thickness	0.41, 0.0001	-2.13, 0.042	4.93, < 0.0001
Capillary luminal area	0.71, < 0.0001	0.81, 0.425	-8.09, < 0.0001

‡Disease status (dummy variable 0 vs. 1): 0 = control, 1 = diabetes (regular biopsy + nerves from amputation)

Table S2. Target sequences of indicated shRNA in the current study

	Target sequence
shCD40#1‡	AGCAGGAGACTGGCTAAATAA
shCD40#2	CTCTTGGTGCTGGTCTTTATC
shCD40#3	ATGCCTTCCTTGCGGTGAAAG
shCD40#4‡	TCCAGTCTCCCAACTTGTATT
shHIF-1α#1	GTGATGAAAGAATTACCGAAT
shHIF-1α#2‡	CCGCTGGAGACACAATCATAT
shHIF-1α#3	TGCTCTTTGTGGTTGGATCTA
shHIF1-α#4‡	CGGCGAAGTAAAGAATCTGAA
shPTEN#1‡	CCACAGCTAGAACTTATCAAA
shPTEN#2	CTAGAACTTATCAAACCCTTT
shPTEN#3	CGTGCAGATAATGACAAGGAA
shPTEN#4‡	CCACAAATGAAGGGATATAAA

‡The knockdown efficiency of these shRNA was higher in each group determined by Western blot analysis.
